# Musculoskeletal disorders in interventional radiology workers: an integrative review

**DOI:** 10.47626/1679-4435-2022-860

**Published:** 2023-08-08

**Authors:** Carolina Neis Machado, Otávio Bitencourt de Freitas, Juliana Almeida Coelho de Melo, Gerusa Ribeiro

**Affiliations:** 1 Departamento Acadêmico de Saúde e Serviços, Instituto Federal de Educação, Ciência e Tecnologia de Santa Catarina, Florianópolis, SC, Brazil

**Keywords:** radiology, interventional, radiation protection, personal protective equipment, occupational diseases, musculoskeletal disorders, radiologia intervencionista, proteção radiológica, equipamento de proteção individual, doenças ocupacionais, distúrbios musculoesqueléticos

## Abstract

This integrative review analyzed scientific production on musculoskeletal disorders related to personal protective equipment used by interventional radiology teams. The PubMed, Embase, and SciELO databases were searched using a strategy developed with the help of a librarian. The double-blind selection process involved the Rayyan online tool. A total of 12 articles were included, which were organized according to year of publication, country research subjects, study type, and main outcomes. Five thematic categories emerged from the analysis: “personal protective equipment”; “ergonomics in the interventional radiology environment”; “the composition of personal protective equipment”; “radiation protection for interventional teams” and “the prevalence of musculoskeletal symptoms in interventional teams”. Outcomes associated with musculoskeletal disorders among interventional teams predominated in the studies, and advances in radiological protection were reported, especially in shielding technologies, as well as continuing efforts toward more ergonomic protective equipment to reduce the risk of musculoskeletal disorders.

## Abstract (pt)

O presente estudo teve como objetivo analisar a produção científica acerca dos distúrbios musculoesqueléticos relacionados ao uso de equipamentos de proteção individual por profissionais da equipe de radiologia intervencionista. Tratou-se de uma revisão integrativa realizada nas bases de dados PubMed, Embase e SciELO. A estratégia de busca foi definida com o auxílio de uma bibliotecária. Os artigos que compuseram a amostra foram selecionados por meio de seleção duplo-cega, utilizando a ferramenta *on-line* Rayyan. Após a aplicação dos critérios de inclusão e exclusão determinados, 12 artigos foram selecionados para o desenvolvimento da presente revisão. Os artigos foram organizados por ano e país de origem, sujeitos da pesquisa, tipo de estudo e principais desfechos. Da análise dos estudos selecionados, emergiram cinco categorias temáticas: “dispositivos de proteção radiológica”; “ergonomia no ambiente de radiologia intervencionista”; “composição dos dispositivos de proteção radiológica”; “proteção radiológica da equipe intervencionista”; e “prevalência de sintomas musculoesqueléticos na equipe intervencionista”. A partir da presente revisão integrativa, foi possível identificar um predomínio de desfechos relacionando o trabalho na área intervencionista com o desenvolvimento de distúrbios musculoesqueléticos, bem como de avanços no campo da proteção radiológica, principalmente em tecnologias de blindagem, tornando-as antropometricamente adequadas para diminuir o risco de distúrbios musculoesqueléticos.

## INTRODUCTION

The widespread use of X-ray radiation requires investigation into the safety and protection of health professionals. The risks involved in its use, especially in interventional procedures, has stimulated the evolution and diversification of personal radiation protection equipment.^1^

Interventional radiology, a unique specialty involving diagnostic, therapeutic, vascular, and non-vascular procedures, is aimed at virtually all patient populations. Currently, most interventional professionals perform several hours-long procedures per day, and units operate 24 hours a day, 7 days a week. The physical demands are different from those of diagnostic radiology in that interventional radiology requires upright posture, heavy personal protective apparel, performing technically complex procedures, and moving equipment and changing positions to perform these procedures.^2^

Lead aprons are one of the most important garments for health professionals who may be exposed to ionizing radiation. Thyroid protectors, gloves, and lead glass goggles further reduce exposure. To shield against ionizing radiation, lead has historically been the most common material due to its great attenuation potential. The use of other materials, such as barium, bismuth, and antimony, is increasing and lead-free or low-lead alloys are now available. Higher lead equivalency increases protection, but makes the equipment heavier.^3^

Due to musculoskeletal injuries, manufacturers of radiological protection equipment have investigated ways of reducing its burden to ensure an adequate balance between comfort and protection, ie, reducing overload without compromising safety. Unfortunately, this is not always achievable. Generally, lead aprons do not achieve the double objective of excellent radiation protection while avoiding ergonomic hazards, since the lighter they are, the farther they fall below acceptable protection standards.^2^

International Electrotechnical Commission standard 61331^4^ addresses general requirements for medical protective equipment in terms of design and the attenuation properties of the materials, but not ergonomics. Although protective apparel can effectively reduce exposure to X-rays, it can cause mechanical overload in users, which could result in negative effects on the musculoskeletal system, especially the spine.^4^

Given the above, it is important to study knowledge production about musculoskeletal disorders related to personal protective apparel among interventional radiology team members. Thus, the aim of this study was to conduct an integrative review on the topic to raise awareness about these disturbances, alternatives for resolving them, and to promote new solutions.

## METHODS

This integrative literature review addressed the following research question: “What is the ergonomic influence of personal protective equipment on interventional radiology workers?” The review’s methodology followed a protocol by Forte et al.^5^ involving the following stages: identifying the theme and formulating the research question, defining the inclusion and exclusion criteria, categorizing studies according to the inclusion and exclusion criteria, evaluating and critically analyzing the findings, and presenting the findings.

The descriptors and keywords, as well as the search strategies, were defined through consultation with a librarian and are detailed in Table 1. The Boolean operators AND and OR were used to maximize the number of results. The SciELO search strategy differed from the PubMed and Embase strategies in that Portuguese and Spanish descriptors and keywords were used, in addition to deleting the theme “occupational health”, to improve the results.

**Table 1:** Study characteristics

No.	Year/country	Authors	Participants	Study type	Objectives
1	2020, Italy	Monaco et al.^6^	Scientific literature	Systematic review	To evaluate the relationship between lead apron use and work-related musculoskeletal diseases.
2	2020, Colombia	Vélez^7^	Scientific literature	Literature review	To investigate protection and safety methods in interventional radiology environments.
3	2018, USA	Benjamin & Meisinger^8^	Scientific literature	Literature review	To identify ergonomic challenges related to interventional radiology and provide specific background, guidelines, and recommendations for musculoskeletal injury prevention.
4	2018, USA	Rees & Duncan^1^	Scientific literature	Literature review	To examine the relationship between protective equipment and musculoskeletal disorders in interventional radiology.
5	2017 USA	Dixon et al.^2^	Scientific literature	Literature review	To investigate occupational disorders of the cervical and lumbar region in interventional physicians.
6	2015, USA	Orme et al.^9^	Interventional cardiology team	Survey	To determine whether the prevalence of work-related musculoskeletal pain, cancer, and other medical conditions is higher among interventional laboratory staff than other staff.
7	2013, Brazil	Flôr & Gelbcke^10^	Hospital hemodynamic care unit	Qualitative study	To analyze nursing staff attitudes about protective measures in interventional radiology procedures.
8	2013, India	Fattal & Goldstein^11^	Operators of interventional radiology equipment	Case report	Clinical experience with a new protection system to eliminate radiation exposure.
9	2011, USA	Marichal et al.^12^	Simulated interventionalist	Comparative study	To compare a suspended protection system with standard lead aprons in a simulated interventionalist.
10	2009, Russia	Klein et al.^13^	Occupational risk data in interventional radiology	Trial	To report the occupational risk prevalence for interventional radiology teams and summarize epidemiological studies designed to determine these risks.
11	2004, Finland	Vehmas^14^	Scientific literature	Literature review	To discuss the role of radiology in occupational medicine and work-related issues in radiology departments.
12	2002, Australia & New Zeland	Rothmore^15^	Interventional physicians	Cross-longitudinal study	To compare discomfort, fatigue, and ease of movement in interventional physicians when using different lead garments.

The inclusion criteria were complete scientific articles published in English or Portuguese that contained the relevant descriptors/keywords in the abstract and/or title and whose general and/or specific objectives explicitly referred to the research question. The time frame, 1990 to 2020, was based on the creation of the National Center for Cardiovascular Interventions (*CENIC*) in 1991. This body created an official database to document the performance and development of interventional radiology in Brazil. We expected there to be an increase in related publications after this milestone.

The exclusion criteria were any other publication format (ie, letters, reviews, theses, dissertations, editorials, books, book chapters, government documents, and newsletters), studies unavailable in full online, publications prior to 1990, and duplicate records.

The PubMed, Embase, and SciELO databases were searched in September 2020. The search was performed using the Federated Academic Community/Federal Institute of Santa Catarina network (CAFe/IFSC). These databases were selected due to being open access (for greater online access to full texts) and conducive to Brazilian and international publications on the theme.

Article selection was performed by 2 researchers (OBF and CNM) who were blinded using Rayyan, an online tool developed by the Qatar Computing Research Institute. The categories of analysis emerged from the content of the included articles.

## RESULTS

**Figure 1:**
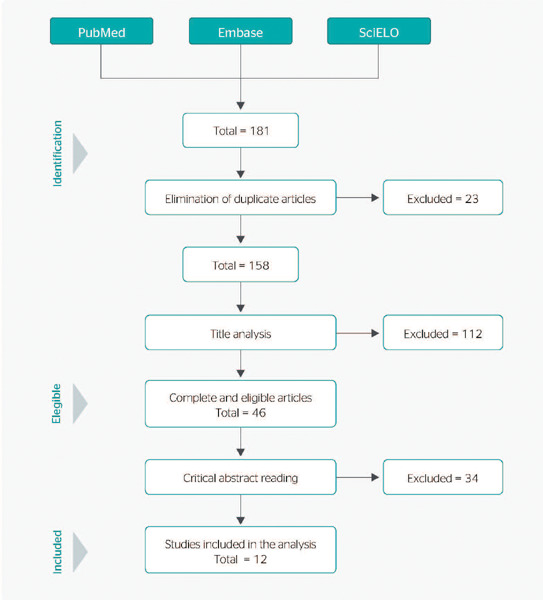
Article selection flowchart.

When the search filters were applied, a total of 181 articles were found in the databases, 23 of which were duplicate records. After reading the titles, 46 articles were selected for abstract analysis, after which 12 were selected for full analysis and categorization. The article selection flowchart is shown in Figure 1. The 12 included studies were organized by year of publication, country, participants, study type, and main outcomes (Table 1).

Regarding the number of included articles, *Techniques in Vascular and Interventional Radiology* and *Journal of Vascular and Interventional Radiology* published 2 each. Rees (USA) and Goldstein (India and Russia) each authored 2 studies. The publications were concentrated in 2020, 2018, and 2013, which demonstrates that, although there has been reflection on this theme for some time, problems persist.

Of the 12 studies included in this review, 6 were literature reviews (narrative, integrative, or systematic), 5 involved field research on the health of interventionist staff, and the other contained epidemiological information about the risk of occupational disease in an interventional team. Regardless of the design, outcomes associated with musculoskeletal disorders among interventional teams predominated.

Of special note was the scarcity of Brazilian studies. A single study was found within the scope of this review. Flôr & Gelbcke^10^ reviewed radiological nursing studies in important national journals, including *Revista Texto e Contexto, Revista Brasileira de Enfermagem, Revista de Enfermagem da Universidade de Pernambuco,* and *Revista de Enfermagem da Universidade do Estado do Rio de Janeiro,* regarding the effects of ionizing radiation and physical load.

## DISCUSSION

Five thematic categories emerged from the analysis: “personal protective equipment”; “ergonomics in the interventional radiology environment”; “the composition of personal protective equipment”; “radiological protection for interventional teams”; and “the prevalence of musculoskeletal symptoms in interventional teams”.

### PERSONAL PROTECTIVE EQUIPMENT

The primary purpose of radiation protection is to reduce or prevent exposure to ionizing radiation. The use of protective equipment is mandatory in the interventional environment to control exposure.^11^ Since it is not optional, in addition to shielding effectiveness, investigations about the equipment’s dimensions and user adaptability are important. Such equipment is worn an average of 5 hours a day, 2-5 days a week.^15^ Rothmore^15^ points out that the correct apparel must be selected and recommends segmented pieces (vest/skirt) due to better mobility. If this is not an option, whole aprons with adjustable waists should be used. Benjamin & Meisinger^8^ report that, in addition to lead shielding, correct apparel types must also be used to maximize effectiveness and provide adequate protection. An inadequate relationship between users and equipment can lead to radiation exposure, injuries, and musculoskeletal pain.^8^

Despite the importance this subject is given in the literature, inattention to personal protective equipment is still frequent. According to Chen et al.,^16^ usage of lead aprons is low, around 70%, and almost 10% of the staff in their sample did not know the correct position for the dosimeter. According to Wilson-Stewart et al.,^17^ non-use or incorrect use of radiation protective equipment could result in exceeding the recommended dose. A 2016 Algerian survey indicated that correct clothing can reduce the annual effective dose to < 10% in interventional cardiologists, while the apron and thyroid protector set further reduces exposure to < 4%.^18^ The authors pointed out that the main error among workers is underuse of protective glasses, which leads to excessive annual doses. They highlighted training and awareness about personal protective equipment, even for professionals outside the radiology department.

### ERGONOMICS IN THE INTERVENTIONAL RADIOLOGY ENVIRONMENT

Although the risks of cumulative exposure to radiation have been studied for decades, some important points have been overlooked. The balance between ergonomics and protection should be improved. Safety equipment must keep pace with the dramatic evolution of imaging technology. As a metaphor for this relationship, Klein et al.^13^ described how technological innovation led to airbags, which have made driving far safer than seat belts alone, with current protective equipment being like seat belts. Although they concede that lead protection is efficient, an ideal equipment design has yet to be commercialized, resulting in widespread user discomfort from the burden. Soares et al.^19^ analyzed anthropometric characteristics and hand grip strength, finding a significant relationship between these two variables, concluding that ergonomic concerns directly influence work quality and the impact of work activities.

However, ergonomics also positively affects the radiological environment. The proper positioning of monitors in fluoroscopy, for example, prevents neck pain. Setting the procedure tables to the correct height alleviates the impact on elbows, reducing overload on the brachialis muscles.^8^ Benjamin & Meisinger highlight strategies for minimizing musculoskeletal damage in very long routines, including keeping physically fit, maintaining good posture at work, alternating foot support using a support bench, and regular stretching.^8^ In a study on nursing staff attitudes about protective measures in interventional radiology procedures, Flôr & Gelbcke^10^ observed complaints of discomfort related to protective apparel use. Corroborating this, Pereira^20^ reported that the location of materials and the weight of lead aprons were ergonomic difficulties in the interventionist environment.

### THE COMPOSITION OF RADIATION PROTECTION EQUIPMENT

Traditionally, lead has been used in radiation protective equipment due to its high attenuation level.^3,6^ However, barium, bismuth, and other materials have been increasingly studied as alternatives to lead. Kang et al.^21^ concluded that personal protective apparel based on urethane resin and bismuth nanopowder provides excellent performance, mainly due to its flexibility.^21^ Schlattl et al.^22^ compared lead alternatives in 3 different X-ray beams (60, 75, and 120 kV), finding that the a minimum effective dose increases 6% for tin and 3% for a combination of tin and bismuth.^22^ Cetin et al.^23^ concluded that, despite being cheap and easily accessible, lead is toxic and heavy. Lead alternatives, including bismuth, tin, antimony, and tungsten, can be used in protective apparel, providing better protection than a 0.25-mm lead garment, while being 85% lighter.

### RADIATION PROTECTION FOR INTERVENTIONAL TEAMS

The interventional radiology environment involves high occupational risk due to long exposure times to ionizing radiation.^24^ Thus, protection against occupational exposure is required for everyone working in the unit. This includes not only radiology technicians, physicians, and nurses, but others who may be there only occasionally, such as anesthesiologists.^24^ These workers require proper monitoring and protective equipment.^14^ Flôr & Gelbcke^10^ reiterate that interventional teams suffer the impact of ionizing radiation without being prepared to minimize it.

Radiation safety measures, such as dosimeters and adherence to the time, distance, and shielding principles, should be encouraged in the work environment, striving toward the “as low as reasonably achievable” concept. Klein et al.^13^ point out that if ergonomics can accompany technological advances in interventional practice, workers will have safer and more comfortable careers. Radiological protection, especially in interventional radiology, requires alignment between ergonomics and high-tech environments.^14^ Attention to occupational health is lacking in interventional medicine.

According to Chen et al., interventional radiology teams have a weak understanding of radiation protection.^16^ König et al. reported that the consequences of prolonged low-dose X-ray exposure are still being studied and remain controversial.^25^ While some current studies report that radiology workers are not at increased risk of malignant diseases,^26,27^ others state that their risk of brain tumors is doubled, in addition to a moderate risk of melanoma and breast cancer.^28^

### PREVALENCE OF MUSCULOSKELETAL SYMPTOMS IN INTERVENTIONAL TEAMS

Concern for the health of interventionists led to the creation of the Multi-Specialty Occupational Health Group in 2005 in the United States. The central objective of this group is to improve the occupational health of interventional teams, determine the impact of occupational conditions, risks on an epidemiological scale, and the dangers mitigated in the laboratory to better direct efforts to minimize exposure.^13^

According to Klein et al.,^13^ there is an association between the use of lead clothing and the back, hip, knee, and ankle pain experienced by a quarter of interventional workers. Another relationship was the association between work hours and back problems among interventional laboratory staff.^18^ Benjamin & Meisinger^8^ pointed out that spine-related complaints exceed 40% among workers with more than 10 years of experience. This number increases to 60% among those with > 20 years of experience. A further 28% report hip, knee, and ankle problems. Approximately 33% of those who reported back pain said they missed work due to symptoms in this region.^8^

Back pain accompanies interventional workers to such a degree that “interventional disc disease” was proposed in the late 1990s.^8^ Benjamin & Meisinger also compared rheumatologists, orthopedists, liver surgeons, and cardiac interventionists, with the latter having a higher incidence of musculoskeletal pain complaints and longer periods of missed work. Another important fact is that most workers who used lead protective apparel had herniated discs.^8^ In contrast, Monaco et al.^6^ failed to find a correlation between protective apparel and musculoskeletal disorders in interventional radiology workers. Despite the more evident discomfort in users, this relationship has not yet been fully established.^6^

## FINAL CONSIDERATIONS

The present review found that shielding power against ionizing radiation has improved in radiation protective equipment between 1990 and 2020. Outcomes related to the development of musculoskeletal disorders predominated in the studies. The manufacture of lighter and more ergonomic apparel has been investigated to reduce the risk of these disorders. The included studies also called for better education about radiation protection.

Although this integrative review revealed a scarcity of Brazilian publications on musculoskeletal disorders in interventional radiology promising advances were also identified in radiation protection and interventional radiology, mainly in shielding technologies.

The present study can alert health professionals, especially teams that work with ionizing radiation and personal protective equipment, to consider their methods and seek alternative strategies of improving occupational health.

## Figures and Tables

**Chart 1:** Databases, filters, and search strategies

Database	Search strategy
PubMed/MEDLINE	((“Radiation Protection” [Mesh] OR “Radiation Protection” OR “Irradiation protection” OR “Radiation prevention” OR “Radio protection” OR “Radioprotection” OR “Personal Protective Equipment” [Mesh] OR “Personal Protective Equipment” OR “Personal Protection Equipment” OR “Personal Protective Equipment”) AND (“Radiology, Interventional” [Mesh] OR “Interventional Radiology” OR “Angiography” [Mesh] OR “Angiography” OR “Angiographies” OR “Angiogram” OR “Angiograms” OR “Arteriography” OR “Arteriographies” OR “Catheterization” [Mesh] OR “Catheterization” OR “Catheterizations” OR “Cannulation” OR “Cannulations”) AND (“Occupational Health” [Mesh] OR “Occupational Health” OR “Occupational Safety” OR “Employee Health”))
Embase	((“Radiation Protection” OR “Irradiation protection” OR “Radiation prevention” OR “Radio protection” OR “Radioprotection” OR “Personal Protective Equipment” OR “Personal Protection Equipment” OR “Personal Protective Equipment”) AND (“Interventional Radiology” OR “Angiography” OR “Angiographies” OR “Angiogram” OR “Angiograms” OR “Arteriography” OR “Arteriographies” OR “Catheterization” OR “Catheterizations” OR “Cannulation” OR “Cannulations”) AND (“Occupational Health” OR “Occupational Safety” OR “Employee Health”))
SciELO	((“Radiation Protection” OR “Irradiation protection” OR “Radiation prevention” OR “Radio protection” OR “Radioprotection” OR “Personal Protective Equipment” OR “Personal Protection Equipment” OR “Personal Protective Equipment” OR “Proteção Radiológica” OR “Proteção contra Radiação” OR “Proteção contra Radiações” OR “Proteção contra a Radiação” OR “Proteção contra as Radiações” OR “Radioproteção” OR “Equipamento de Proteção Individual” OR “Equipamentos de Proteção Individual” OR “Equipamento de Proteção Pessoal” OR “Equipamentos de Proteção Pessoal” OR “Protección Radiológica” OR “Protección contra Radiaciones” OR “Protección contra Radiación” OR “Protección contra la Radiación” OR “Protección contra las Radiaciones” OR “Radioprotección” OR “Equipo de Protección Personal” OR “Equipos de Protección Personal”) AND (“Interventional Radiology” OR “Angiography” OR “Angiographies” OR “Angiogram” OR “Angiograms” OR “Arteriography” OR “Arteriographies” OR “Catheterization” OR “Catheterizations” OR “Cannulation” OR “Cannulations” OR “Radiologia Intervencionista” OR “Angiografia” OR “Angiograma” OR “Arteriografia” OR “Cateterismo” OR “Canulação” OR “Cateterização” OR “Radiología Intervencionai” OR “Canulación” OR “Cateterización”))

## References

[1] Rees CR, Duncan BWC (2018). Get the lead off our backs!. Tech Vasc Interv Radiol.

[2] Dixon RG, Khiatani V, Statler JD, Walser EM, Midia M, Miller DL (2017). Society of Interventional Radiology: Occupational back and neck pain and
the interventional radiologist. J Vasc Interv Radiol.

[3] Bartal G, Sailer AM, Vano E (2018). Should we keep the lead in the aprons?. Tech Vasc Interv Radiol.

[4] IEC (2014). IEC 61331-3:2014 Protective Devices Against Diagnostic Medical X-Radiation – Part
3: Protective Clothing, Eyewear and Protective Patient Shields.

[5] Forte ECN, Medeiros F, Pires DEP (2013). Protocolo de revisão integrativa de literatura sobre a
satisfação no trabalho dos enfermeiros/as da Atenção
Primária em Saúde.

[6] Monaco MGL, Carta A, Tamhid T, Porru S (2020). Anti-X apron wearing and musculoskeletal problems among healthcare workers:
a systematic scoping review. Int J Environ Res Public Health.

[7] Vélez MJ (2020). Riesgos osteomusculares: patología ortopédica en el
cardiólogo intervencionista. Rev Colomb Cardiol.

[8] Benjamin JL, Meisinger QC (2018). Ergonomics in the development and prevention of musculoskeletal injury in
interventional radiologists. Tech Vasc Interv Radiol.

[9] Orme NM, Rihal CS, Gulati R, Holmes DR Jr., Lennon RJ, Lewis BR (2015). Occupational health hazards of working in the interventional laboratory: a
multisite case control study of physicians and allied staff. J Am Coll Cardiol.

[10] Flôr RC, Gelbcke FL (2013). Proteção radiológica e a atitude de trabalhadores de
enfermagem em serviço de hemodinâmica. Texto Contexto Enferm.

[11] Fattal P, Goldstein JA (2013). A novel complete radiation protection system eliminates physician radiation
exposure and leaded aprons. Catheter Cardiovasc Interv.

[12] Marichal DA, Anwar T, Kirsch D, Clements J, Carlson L, Savage C (2011). Comparison of a suspended radiation protection system versus standard lead
apron for radiation exposure of a simulated interventionalist. J Vasc Interv Radiol.

[13] Klein LW, Miller DL, Balter S, Laskey W, Haines D, Norbash A (2009). Occupational health hazards in the interventional laboratory: time for a
safer environment. Radiology.

[14] Vehmas T (2004). Role of radiology in occupational medicine. Acta Radiol.

[15] Rothmore P (2002). Lead aprons, radiographers and discomfort: a pilot study. J Occup Health Saf Aust N Z.

[16] Chen X, Zhang R, Lai M, Yang S, Yang C (2017). Radiation protection capability and personal protection in interventional
radiology: Current situation in grade-Ill hospital. J Interv (China).

[17] Wilson-Stewart K, Shanahan M, Fontanarosa D, Davidson R (2018). Occupational radiation exposure to nursing staff during cardiovascular
fluoroscopic procedures: A review of the literature. J Appl Clin Med Phys.

[18] Khelassi-Toutaoui N, Toutaoui A, Merad A, Sakhri-Brahimi Z, Baggoura B, Mansouri B (2016). Assessment of radiation protection of patients and staff in interventional
procedures in four Algerian hospitals. Radiat Prot Dosimetry.

[19] Soares AV, Carvalho Júnior JM, Carvalho AM, Martignago RB, Domenech SC, Borges Júnior NG (2015). Relações entre a força de preensão e aspectos
antropométricos da mão. Rev Bras Med Trab.

[20] Pereira AG (2015). O profissional de enfermagem no serviço de hemodinâmica na
perspectiva da ergonomia e proteção radiológica.

[21] Kang JH, Oh SH, Oh Jl, Kim SH, Choi YS, Hwang EH (2019). Protection evaluation of non-lead radiation-shielding fabric: preliminary
exposure-dose study. Oral Radiol.

[22] Schlattl H, Zankl M, Eder H, Hoeschen C (2007). Shielding properties of lead-free protective clothing and their impact on
radiation doses. Med Phys.

[23] Cetin H, Yurt A, Yuksel SH (2017). The absorption properties of lead-free garments for use in radiation
protection. Radiat Prot Dosimetry.

[24] Miller DL, Vano E, Bartal G, Balter S, Dixon R, Padovani R (2010). Occupational radiation protection in interventional radiology: a joint
guideline of the Cardiovascular and Interventional Radiology Society of Europe and the
Society of Interventional Radiology. Cardiovasc Intervent Radiol.

[25] König AM, Etzel R, Thomas RP, Mahnken AH (2019). Personal radiation protection and corresponding dosimetry in interventional
radiology: an overview and future developments. Rofo.

[26] Kitahara CM, Linet MS, Balter S, Miller DL, Rajaraman P, Cahoon EK (2017). Occupational radiation exposure and deaths from malignant intracranial
neoplasms of the brain and CNS in U.S. Radiologic Technologists,
1983-2012. AJR Am J Roentgenol.

[27] Linet MS, Hauptmann M, Freedman DM, Alexander BH, Miller J, Sigurdson AJ (2006). Interventional radiography and mortality risks in U.S. radiologic
technologists. Pediatr Radiol.

[28] Rajaraman P, Doody MM, Yu CL, Preston DL, Miller JS, Sigurdson AJ (2016). Cancer risks in U.S. radiologic technologists working with fluoroscopically
guided interventional procedures, 1994-2008. AJR Am J Roentgenol.

